# Treatment to Be Persued in the Admission of Air into the Veins

**Published:** 1845-12

**Authors:** 


*On the Treatment to be pursued in the Admission of Air into the Veins.*—The plan here mentioned is one given in a monograph by Dr. Wattman, upon the subject of the admission of air into the veins, and is said by the author to be a certain means of preventing the fatal consequences.

The plan consists in the instant closure of the orifice in the vein by the finger, and dashing cold water into the face, if the patient has already experienced symptoms of delirium. The permanent closure of the vein is to be effected, either by gently closing and uniting the lips of the wound, or by ligature or torsion.—*Am. Jour. Med. Sci.*—*Ibid.*

